# 4-(4-Amino-2-fluoro­phen­oxy)-7-meth­oxy­quinazolin-6-ol methanol monosolvate

**DOI:** 10.1107/S1600536812011725

**Published:** 2012-03-24

**Authors:** Wei Huang, Aimin Tan

**Affiliations:** a Jiangsu Key Laboratory of Molecular Targeted Antitumor Drug Research, Jiangsu Simcere Pharmaceutical R&D Co. Ltd, Nanjing 210042, People’s Republic of China; b Institute of Functional Biomolecules, State Key Laboratory of Pharmaceutical Biotechnology, Nanjing University, Nanjing 210093, People’s Republic of China

## Abstract

In the title compound, C_15_H_12_FN_3_O_3_·CH_3_OH, the dihedral angle between the quinazoline ring system and the benzene ring is 81.18 (9)°. In the crystal, mol­ecules are linked by N—H⋯O and O—H⋯N hydrogen bonds, generating [10-1] chains of alternating main mol­ecules and solvent mol­ecules. Weak C—H⋯O inter­actions are also observed.

## Related literature

For background to quinazolinones, see: Priya *et al.* (2011 ▶). For further synthetic details, see: Furuta *et al.* (2006 ▶).
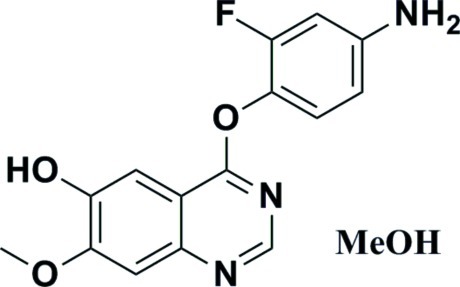


## Experimental

### 

#### Crystal data

C_15_H_12_FN_3_O_3_·CH_4_O
*M* *_r_* = 333.32
Triclinic, 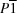

*a* = 8.723 (2) Å
*b* = 8.921 (2) Å
*c* = 11.500 (3) Å
α = 70.925 (4)°
β = 69.940 (4)°
γ = 77.273 (4)°
*V* = 788.6 (3) Å^3^
*Z* = 2
Mo *K*α radiation
μ = 0.11 mm^−1^
*T* = 298 K
0.15 × 0.12 × 0.10 mm

#### Data collection

Bruker SMART 4K CCD diffractometer
Absorption correction: multi-scan (*SADABS*; Bruker, 2001 ▶) *T*_min_ = 0.974, *T*_max_ = 0.989
4819 measured reflections
2747 independent reflections
2010 reflections with *I* > 2σ(*I*)
*R*_int_ = 0.030

#### Refinement

*R*[*F* ^2^ > 2σ(*F* ^2^)] = 0.085
*wR*(*F* ^2^) = 0.193
*S* = 1.17
2747 reflections
227 parameters
2 restraints
H atoms treated by a mixture of independent and constrained refinement
Δρ_max_ = 0.22 e Å^−3^
Δρ_min_ = −0.31 e Å^−3^

### 

Data collection: *SMART* (Bruker, 2001 ▶); cell refinement: *SAINT* (Bruker, 2001 ▶); data reduction: *SAINT*; program(s) used to solve structure: *SHELXS97* (Sheldrick, 2008 ▶); program(s) used to refine structure: *SHELXL97* (Sheldrick, 2008 ▶); molecular graphics: *PLATON* (Spek, 2009 ▶); software used to prepare material for publication: *SHELXTL* (Sheldrick, 2008 ▶).

## Supplementary Material

Crystal structure: contains datablock(s) global, I. DOI: 10.1107/S1600536812011725/hb6683sup1.cif

Structure factors: contains datablock(s) I. DOI: 10.1107/S1600536812011725/hb6683Isup2.hkl

Supplementary material file. DOI: 10.1107/S1600536812011725/hb6683Isup3.cml

Additional supplementary materials:  crystallographic information; 3D view; checkCIF report

## Figures and Tables

**Table 1:** Hydrogen-bond geometry (Å, °)

*D*—H⋯*A*	*D*—H	H⋯*A*	*D*⋯*A*	*D*—H⋯*A*
O2—H2⋯N3^i^	0.82	1.98	2.799 (4)	172
N3—H3*A*⋯O1^ii^	0.85 (2)	2.60 (4)	3.192 (4)	128 (4)
N3—H3*B*⋯O5^iii^	0.88 (2)	2.08 (2)	2.953 (6)	173 (4)
O5—H5⋯N1	0.82	1.95	2.765 (4)	177
C15—H15⋯O5^iv^	0.93	2.57	3.453 (5)	159

Symmetry codes: (i) 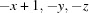; (ii) 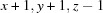; (iii) 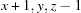; (iv) 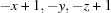.

**Fig. 1.**
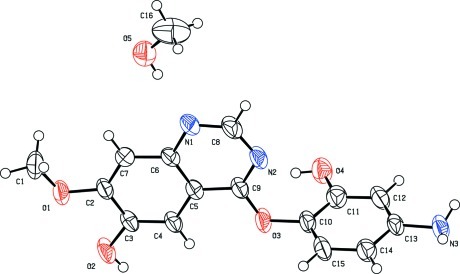
View of the molecule of (I) showing displacement ellipsoids drawn at the 50% probability level.

**Fig. 2.**
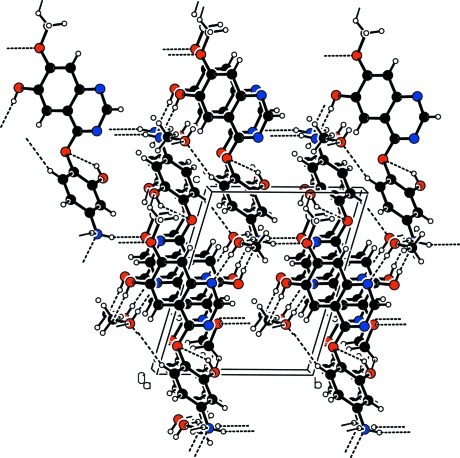
The packing of (I) with hydrogen bonds drawn as dashed lines.

## supplementary crystallographic information

### Comment

4(3*H*)-Quinazolinones are an important class of fused heterocycles with different biological properties such as anti-fungal activities (Priya *et al.*, 2011).

The title compound, (I), is a 4(3*H*)-Quinazolinones intermediate for the synthesis of kinase inhibitor. We present here the structure of the title compound (I), C_15_H_12_FN_3_O_3_.CH_3_OH, crystallized as a methanol solvate (Figure 1).

The bicyclic quinazoline system is effectively planar, with a mean deviation from planarity of 0.0190 (3)°.The quinazoline heterocyclic system and the adjacent benzene ring make a dihedral angle of 81.18 (9)°. In the crystal, the molecules are linked *via* the methanol solvent molecule through O—H_ _ _N,*N*—H_ _ _O and C—H_ _ _O hydrogen bonds(Table 1), so forming chains propagating along the *b* axis direction as shown in Fig. 2.

### Experimental

The title compound was synthesized according to the literature method (Furuta *et al.*, 2006). Colourless blocks were grown from dichloromethane at 277 K.

### Refinement

The H2 atom on O2 and H5 on O5 were located in a difference Fourier map and refined freely with an isotropic temperature factors. All other H-atoms were positioned geometrically and refined using a riding model with d(C—H) = 0.93 Å, *U*_iso_=1.2*U*_eq_ (C) for aromatic and 0.96 Å, *U*_iso_ = 1.5*U*_eq_ (C) for CH_3_ atoms.

### Crystal data

**Table:**

C_15_H_12_FN_3_O_3_·CH_4_O	*Z* = 2
*M**_r_* = 333.32	*F*(000) = 348
Triclinic, *P*1	*D*_x_ = 1.404 Mg m^−^^3^
Hall symbol: -P 1	Mo *K*α radiation, λ = 0.71073 Å
*a* = 8.723 (2) Å	Cell parameters from 2516 reflections
*b* = 8.921 (2) Å	θ = 2.6–25.3°
*c* = 11.500 (3) Å	µ = 0.11 mm^−^^1^
α = 70.925 (4)°	*T* = 298 K
β = 69.940 (4)°	Block, colorless
γ = 77.273 (4)°	0.15 × 0.12 × 0.10 mm
*V* = 788.6 (3) Å^3^	

### Data collection

**Table:**

Bruker SMART 4K CCD diffractometer	2747 independent reflections
Radiation source: fine-focus sealed tube	2010 reflections with *I* > 2σ(*I*)
Graphite monochromator	*R*_int_ = 0.030
φ and ω scans	θ_max_ = 25.0°, θ_min_ = 2.0°
Absorption correction: multi-scan (*SADABS*; Bruker, 2001)	*h* = −10→7
*T*_min_ = 0.974, *T*_max_ = 0.989	*k* = −10→10
4819 measured reflections	*l* = −13→13

### Refinement

**Table:**

Refinement on *F*^2^	Primary atom site location: structure-invariant direct methods
Least-squares matrix: full	Secondary atom site location: difference Fourier map
*R*[*F*^2^ > 2σ(*F*^2^)] = 0.085	Hydrogen site location: inferred from neighbouring sites
*wR*(*F*^2^) = 0.193	H atoms treated by a mixture of independent and constrained refinement
*S* = 1.17	*w* = 1/[σ^2^(*F*_o_^2^) + (0.067*P*)^2^ + 0.5658*P*] where *P* = (*F*_o_^2^ + 2*F*_c_^2^)/3
2747 reflections	(Δ/σ)_max_ < 0.001
227 parameters	Δρ_max_ = 0.22 e Å^−^^3^
2 restraints	Δρ_min_ = −0.31 e Å^−^^3^

### Special details

**Table:**

Geometry. All e.s.d.'s (except the e.s.d. in the dihedral angle between two l.s. planes) are estimated using the full covariance matrix. The cell e.s.d.'s are taken into account individually in the estimation of e.s.d.'s in distances, angles and torsion angles; correlations between e.s.d.'s in cell parameters are only used when they are defined by crystal symmetry. An approximate (isotropic) treatment of cell e.s.d.'s is used for estimating e.s.d.'s involving l.s. planes.
Refinement. Refinement of *F*^2^ against ALL reflections. The weighted *R*-factor *wR* and goodness of fit *S* are based on *F*^2^, conventional *R*-factors *R* are based on *F*, with *F* set to zero for negative *F*^2^. The threshold expression of *F*^2^ > σ(*F*^2^) is used only for calculating *R*-factors(gt) *etc*. and is not relevant to the choice of reflections for refinement. *R*-factors based on *F*^2^ are statistically about twice as large as those based on *F*, and *R*- factors based on ALL data will be even larger.

### Fractional atomic coordinates and isotropic or equivalent isotropic displacement parameters (Å^2^)

**Table:**

	*x*	*y*	*z*	*U*_iso_*/*U*_eq_	
C1	0.0141 (7)	−0.2778 (6)	0.8438 (4)	0.0716 (14)	
H1A	0.1224	−0.2790	0.8480	0.107*	
H1B	−0.0365	−0.3654	0.9105	0.107*	
H1C	−0.0510	−0.1790	0.8554	0.107*	
C2	0.1142 (4)	−0.1900 (4)	0.6163 (3)	0.0403 (9)	
C3	0.1429 (4)	−0.2237 (4)	0.4980 (3)	0.0388 (9)	
C4	0.2366 (4)	−0.1341 (4)	0.3861 (3)	0.0394 (9)	
H4	0.2569	−0.1582	0.3089	0.047*	
C5	0.3027 (4)	−0.0040 (4)	0.3884 (3)	0.0365 (8)	
C6	0.2702 (4)	0.0325 (4)	0.5041 (3)	0.0378 (8)	
C7	0.1759 (5)	−0.0628 (4)	0.6196 (4)	0.0438 (9)	
H7	0.1556	−0.0400	0.6973	0.053*	
C8	0.4216 (5)	0.2396 (4)	0.4001 (4)	0.0524 (11)	
H8	0.4621	0.3259	0.4030	0.063*	
C9	0.4066 (5)	0.0943 (4)	0.2791 (3)	0.0401 (9)	
C10	0.5608 (5)	0.1413 (4)	0.0612 (3)	0.0424 (9)	
C11	0.5111 (5)	0.2878 (5)	−0.0120 (4)	0.0482 (10)	
C12	0.6172 (6)	0.3746 (4)	−0.1182 (4)	0.0497 (10)	
H12	0.5798	0.4740	−0.1652	0.060*	
C13	0.7798 (5)	0.3131 (4)	−0.1545 (3)	0.0424 (9)	
C14	0.8321 (5)	0.1640 (4)	−0.0821 (4)	0.0485 (10)	
H14	0.9418	0.1211	−0.1057	0.058*	
C15	0.7208 (5)	0.0794 (4)	0.0251 (4)	0.0498 (10)	
H15	0.7563	−0.0205	0.0725	0.060*	
C16	0.3354 (6)	0.3722 (7)	0.7115 (6)	0.0910 (18)	
H16A	0.3020	0.4117	0.7859	0.136*	
H16B	0.3274	0.4594	0.6373	0.136*	
H16C	0.4471	0.3223	0.6983	0.136*	
O1	0.0249 (3)	−0.2927 (3)	0.7224 (2)	0.0518 (7)	
O2	0.0736 (4)	−0.3501 (3)	0.5054 (2)	0.0569 (8)	
H2	0.0888	−0.3564	0.4326	0.085*	
O3	0.4459 (3)	0.0561 (3)	0.1677 (2)	0.0536 (8)	
N1	0.3312 (4)	0.1598 (3)	0.5098 (3)	0.0499 (9)	
N2	0.4647 (4)	0.2147 (3)	0.2832 (3)	0.0479 (8)	
F1	0.3505 (3)	0.3488 (3)	0.0233 (3)	0.0773 (8)	
N3	0.8919 (5)	0.3961 (4)	−0.2678 (3)	0.0548 (10)	
H3A	0.864 (6)	0.496 (3)	−0.282 (5)	0.082*	
H3B	0.991 (3)	0.355 (5)	−0.261 (4)	0.066*	
O5	0.2333 (4)	0.2605 (4)	0.7305 (3)	0.0699 (9)	
H5	0.2582	0.2309	0.6653	0.105*	

### Atomic displacement parameters (Å^2^)

**Table:**

	*U*^11^	*U*^22^	*U*^33^	*U*^12^	*U*^13^	*U*^23^
C1	0.101 (4)	0.068 (3)	0.035 (2)	−0.042 (3)	0.003 (2)	−0.002 (2)
C2	0.043 (2)	0.0290 (18)	0.041 (2)	−0.0122 (16)	−0.0065 (17)	−0.0001 (16)
C3	0.046 (2)	0.0293 (17)	0.042 (2)	−0.0159 (16)	−0.0149 (17)	−0.0015 (15)
C4	0.047 (2)	0.0310 (18)	0.041 (2)	−0.0167 (16)	−0.0129 (17)	−0.0031 (16)
C5	0.039 (2)	0.0284 (17)	0.040 (2)	−0.0107 (15)	−0.0104 (16)	−0.0033 (15)
C6	0.044 (2)	0.0274 (17)	0.043 (2)	−0.0109 (15)	−0.0117 (17)	−0.0071 (16)
C7	0.050 (2)	0.038 (2)	0.040 (2)	−0.0116 (17)	−0.0039 (18)	−0.0127 (17)
C8	0.074 (3)	0.039 (2)	0.052 (3)	−0.030 (2)	−0.017 (2)	−0.0094 (19)
C9	0.049 (2)	0.0317 (18)	0.039 (2)	−0.0157 (16)	−0.0127 (17)	−0.0012 (15)
C10	0.057 (3)	0.038 (2)	0.033 (2)	−0.0257 (19)	−0.0084 (18)	−0.0030 (16)
C11	0.050 (3)	0.049 (2)	0.046 (2)	−0.014 (2)	−0.0107 (19)	−0.0125 (19)
C12	0.071 (3)	0.0332 (19)	0.045 (2)	−0.021 (2)	−0.019 (2)	0.0018 (17)
C13	0.063 (3)	0.0354 (19)	0.034 (2)	−0.0293 (19)	−0.0111 (19)	−0.0042 (16)
C14	0.057 (3)	0.044 (2)	0.044 (2)	−0.0181 (19)	−0.0113 (19)	−0.0076 (18)
C15	0.063 (3)	0.036 (2)	0.043 (2)	−0.0171 (19)	−0.014 (2)	0.0046 (17)
C16	0.074 (4)	0.095 (4)	0.140 (5)	−0.002 (3)	−0.049 (4)	−0.065 (4)
O1	0.0629 (18)	0.0458 (15)	0.0390 (15)	−0.0292 (13)	−0.0019 (13)	−0.0005 (12)
O2	0.086 (2)	0.0451 (15)	0.0448 (16)	−0.0451 (15)	−0.0136 (16)	−0.0002 (13)
O3	0.075 (2)	0.0495 (15)	0.0355 (14)	−0.0423 (14)	−0.0008 (13)	−0.0039 (12)
N1	0.064 (2)	0.0368 (17)	0.051 (2)	−0.0236 (16)	−0.0090 (17)	−0.0105 (15)
N2	0.064 (2)	0.0373 (17)	0.0438 (19)	−0.0303 (16)	−0.0088 (16)	−0.0040 (14)
F1	0.0647 (18)	0.0707 (17)	0.0825 (19)	−0.0111 (13)	−0.0131 (15)	−0.0101 (14)
N3	0.075 (3)	0.048 (2)	0.0408 (19)	−0.037 (2)	−0.0098 (19)	0.0006 (17)
O5	0.096 (2)	0.0598 (18)	0.0517 (18)	−0.0249 (18)	−0.0110 (17)	−0.0134 (15)

### Geometric parameters (Å, º)

**Table:**

C1—O1	1.416 (5)	C10—C15	1.356 (5)
C1—H1A	0.9600	C10—C11	1.373 (5)
C1—H1B	0.9600	C10—O3	1.403 (4)
C1—H1C	0.9600	C11—F1	1.357 (5)
C2—O1	1.366 (4)	C11—C12	1.368 (5)
C2—C7	1.375 (5)	C12—C13	1.374 (6)
C2—C3	1.415 (5)	C12—H12	0.9300
C3—O2	1.360 (4)	C13—C14	1.393 (5)
C3—C4	1.363 (5)	C13—N3	1.420 (5)
C4—C5	1.416 (4)	C14—C15	1.388 (5)
C4—H4	0.9300	C14—H14	0.9300
C5—C6	1.393 (5)	C15—H15	0.9300
C5—C9	1.421 (5)	C16—O5	1.396 (5)
C6—N1	1.385 (4)	C16—H16A	0.9600
C6—C7	1.409 (5)	C16—H16B	0.9600
C7—H7	0.9300	C16—H16C	0.9600
C8—N1	1.303 (5)	O2—H2	0.8200
C8—N2	1.346 (5)	N3—H3A	0.848 (19)
C8—H8	0.9300	N3—H3B	0.879 (19)
C9—N2	1.304 (4)	O5—H5	0.8200
C9—O3	1.344 (4)		
			
O1—C1—H1A	109.5	C11—C10—O3	120.1 (4)
O1—C1—H1B	109.5	F1—C11—C12	118.9 (4)
H1A—C1—H1B	109.5	F1—C11—C10	118.6 (3)
O1—C1—H1C	109.5	C12—C11—C10	122.5 (4)
H1A—C1—H1C	109.5	C11—C12—C13	119.1 (4)
H1B—C1—H1C	109.5	C11—C12—H12	120.4
O1—C2—C7	124.3 (3)	C13—C12—H12	120.4
O1—C2—C3	115.2 (3)	C12—C13—C14	119.1 (3)
C7—C2—C3	120.5 (3)	C12—C13—N3	120.7 (4)
O2—C3—C4	123.6 (3)	C14—C13—N3	120.1 (4)
O2—C3—C2	115.6 (3)	C15—C14—C13	120.1 (4)
C4—C3—C2	120.7 (3)	C15—C14—H14	119.9
C3—C4—C5	119.4 (3)	C13—C14—H14	119.9
C3—C4—H4	120.3	C10—C15—C14	120.5 (4)
C5—C4—H4	120.3	C10—C15—H15	119.7
C6—C5—C4	120.0 (3)	C14—C15—H15	119.7
C6—C5—C9	115.4 (3)	O5—C16—H16A	109.5
C4—C5—C9	124.6 (3)	O5—C16—H16B	109.5
N1—C6—C5	121.7 (3)	H16A—C16—H16B	109.5
N1—C6—C7	118.2 (3)	O5—C16—H16C	109.5
C5—C6—C7	120.1 (3)	H16A—C16—H16C	109.5
C2—C7—C6	119.2 (3)	H16B—C16—H16C	109.5
C2—C7—H7	120.4	C2—O1—C1	116.9 (3)
C6—C7—H7	120.4	C3—O2—H2	109.5
N1—C8—N2	129.2 (3)	C9—O3—C10	117.4 (3)
N1—C8—H8	115.4	C8—N1—C6	114.9 (3)
N2—C8—H8	115.4	C9—N2—C8	115.3 (3)
N2—C9—O3	120.2 (3)	C13—N3—H3A	111 (3)
N2—C9—C5	123.5 (3)	C13—N3—H3B	106 (3)
O3—C9—C5	116.3 (3)	H3A—N3—H3B	119 (5)
C15—C10—C11	118.6 (3)	C16—O5—H5	109.5
C15—C10—O3	121.3 (3)		
			
O1—C2—C3—O2	2.0 (5)	O3—C10—C11—C12	−178.7 (3)
C7—C2—C3—O2	−178.7 (3)	F1—C11—C12—C13	−179.8 (3)
O1—C2—C3—C4	−177.1 (3)	C10—C11—C12—C13	0.6 (6)
C7—C2—C3—C4	2.2 (5)	C11—C12—C13—C14	0.0 (5)
O2—C3—C4—C5	179.7 (3)	C11—C12—C13—N3	176.8 (3)
C2—C3—C4—C5	−1.3 (5)	C12—C13—C14—C15	0.0 (5)
C3—C4—C5—C6	−0.8 (5)	N3—C13—C14—C15	−176.9 (3)
C3—C4—C5—C9	177.7 (4)	C11—C10—C15—C14	1.2 (6)
C4—C5—C6—N1	−179.3 (3)	O3—C10—C15—C14	178.6 (3)
C9—C5—C6—N1	2.1 (5)	C13—C14—C15—C10	−0.6 (6)
C4—C5—C6—C7	2.0 (5)	C7—C2—O1—C1	−8.3 (6)
C9—C5—C6—C7	−176.5 (3)	C3—C2—O1—C1	170.9 (4)
O1—C2—C7—C6	178.3 (3)	N2—C9—O3—C10	5.3 (5)
C3—C2—C7—C6	−0.9 (5)	C5—C9—O3—C10	−174.0 (3)
N1—C6—C7—C2	−179.9 (3)	C15—C10—O3—C9	99.6 (4)
C5—C6—C7—C2	−1.2 (5)	C11—C10—O3—C9	−83.0 (4)
C6—C5—C9—N2	−2.3 (5)	N2—C8—N1—C6	−0.7 (7)
C4—C5—C9—N2	179.2 (4)	C5—C6—N1—C8	−0.8 (5)
C6—C5—C9—O3	176.9 (3)	C7—C6—N1—C8	177.9 (4)
C4—C5—C9—O3	−1.6 (5)	O3—C9—N2—C8	−178.1 (4)
C15—C10—C11—F1	179.2 (3)	C5—C9—N2—C8	1.0 (6)
O3—C10—C11—F1	1.7 (5)	N1—C8—N2—C9	0.5 (7)
C15—C10—C11—C12	−1.2 (6)		

### Hydrogen-bond geometry (Å, º)

**Table:**

*D*—H···*A*	*D*—H	H···*A*	*D*···*A*	*D*—H···*A*
O2—H2···N3^i^	0.82	1.98	2.799 (4)	172
N3—H3*A*···O1^ii^	0.85 (2)	2.60 (4)	3.192 (4)	128 (4)
N3—H3*B*···O5^iii^	0.88 (2)	2.08 (2)	2.953 (6)	173 (4)
O5—H5···N1	0.82	1.95	2.765 (4)	177
C15—H15···O5^iv^	0.93	2.57	3.453 (5)	159

Symmetry codes: (i) −*x*+1, −*y*, −*z*; (ii) *x*+1, *y*+1, *z*−1; (iii) *x*+1, *y*, *z*−1; (iv) −*x*+1, −*y*, −*z*+1.
